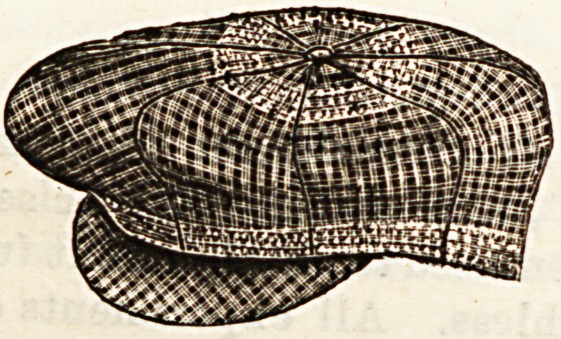# New Appliances and Things Medical

**Published:** 1896-08-15

**Authors:** 


### NEW APPLIANCES AND THINGS MEDICAL.

[We shall be glad to receive, at our Office, 428, Strand, London, W.C., from the manufacturers, specimens of all new preparations and appliances, which may be brought out from time to time.]

### THE "VENTAIR" CAP.

(Ingle and Co., 20, Cheapside, London, E.C.)

Caps are now so universally worn, despite the oppressive feeling which the ordinary description frequently causes the wearer, that attempts of various kinds have often been made to secure thorough ventilation, but so far with indifferent success. The inventors of the "Ventair" cap have solved the difficulty by an ingenious contrivance which enables the material used to be so woven that it affords complete ventilation to the head of the wearer, and adds much to his comfort. The system is simple and efficient. The accompanying block gives a view of one of these caps pulled out of shape, showing the woven ventilators round the top and bottom at the place where the cap fits the head closely. We have tested the "Ventair" cap and have found the system admirable and completely successful. It is always comfortable, the head is kept delightfully cool without any perceptible draught, and the cap is so constructed as to afford additional protection from the sun. The cap is waterproof, being made of "Harris" wools. It is attractive to the eye and hygienically perfect. We are confident that anyone who wears the "Ventair" cap will prefer it to all others. There would be fewer headaches and much more enjoyment from constant outdoor exercise if the "Ventair" cap were to be worn by everybody.

### THE MEMONITOR.

(W. Webber and Co., Plymouth.)

This is a really useful and ingenious contrivance, whereby letters and papers constantly required may be kept together and easily referred to. In form this "Memonitor" is an upright case containing forty-four cards, which enable the papers or letters placed therein to be sub-divided according to the date on which they may be required, or the subject-matter; or if needed for permanent reference they may be placed in the division provided for that purpose. This invention is wonderfully simple, yet thoroughly effectual, and should prove an incalculable blessing to the busy man who can be sure, when placing a letter in its place in this case, of being able to find it immediately it is required.

## Figures and Tables

**Figure f1:**